# Note from the editors: Don’t stop thinking about tomorrow

**DOI:** 10.2807/1560-7917.ES.2020.25.1.2001091

**Published:** 2020-01-09

**Authors:** Eurosurveillance editorial team

**Affiliations:** 1 European Centre for Disease Prevention and Control (ECDC), Stockholm, Sweden

As we are now starting a new decade and thinking about tomorrow, we would like to take stock of selected events in 2019.

One memorable event was the worldwide movement of young people for the climate. Even if not directly related to infectious disease prevention and control, it reminded us of our collective obligation to the upcoming generations. Disease prevention is about improving future health outcomes. Continuous vigilance and surveillance are necessary elements in ensuring the best possible protection of the heath of the population as a whole. Pathogens continue to evolve in their ability to cause human disease. This was demonstrated by the recent detection of pneumonia cases of unknown origin in Wuhan, China since mid-December, for which an unknown, novel corona virus has been preliminarily determined as the cause [1,2]. An example of how pathogens are able to escape tools and technologies developed to control and stop their spread is antimicrobial resistance (AMR). AMR has been a worldwide concern for some time already and has featured prominently in *Eurosurveillance*. In 2019, we covered various aspects of AMR such as the emergence of multidrug-resistant *Neisseria gonorrhoeae* related to international travel [3], an outbreak of extensively drug-resistant *Klebsiella pneumoniae* in a German hospital [4], and the surveillance of antimicrobial consumption and prescribing for example in Belgium, Israel and Switzerland [5-7]. Another aspect driving spread and severity of communicable diseases is human behaviour. The continued spread of measles in Europe over the past decade has been facilitated by insufficient vaccine coverage in specific population groups or regions. Over the past year, vaccine hesitancy and mandatory vaccination were covered in several *Eurosurveillance* articles [8] as were interventions to improve confidence in vaccines, for example, motivational interviews conducted with parents of newborns on maternity wards in Canada resulted in lower hesitancy and greater intention to vaccinate [9].

As usual, food- and waterborne diseases, HIV/AIDS and blood-borne diseases, emerging and vector-borne diseases, influenza and other respiratory diseases were the subject of several articles in the categories, ‘Outbreak reports’ and ‘Surveillance articles’. These two article types have different structures and collectively replaced the former category ‘Surveillance and outbreak reports’ at the beginning of 2019. The associated new instructions for authors have led to greater consistency and completeness of information in submissions in these article categories.

A special issue published in January 2019 illustrated *How advanced diagnostics support public health policy development* [10]. We also published articles on less frequently encountered topics such as a skin rash outbreak traced to a portable floating tank [11] and urban brown rats as the possible source of multidrug-resistant Enterobacteriaceae and meticillin-resistant *Staphylococcus* [12].

In 2019, we published 220 articles (65 rapid communications, 155 regular articles and 21 other items such as editorials, letters and meeting reports). The 2019 acceptance rate of 25% was similar to previous years. As a reflection of the European focus of *Eurosurveillance*, the vast majority of published articles were from Europe, even though 15% of accepted articles in 2019 were from non-European countries. We will continue to welcome contributions from around the globe that are relevant for public health in Europe in the year ahead. Our rapid communications on ongoing or emerging threats were published within 2 to 3 weeks of submission, creating awareness and providing evidence to support rapid public health action. Some examples of the rapids we published included the worsening epidemiological situation of carbapenemase-producing Enterobacteriaceae in Europe [13], a case of extensively drug-resistant typhoid fever imported from Pakistan to Denmark [14] and the detection of a *Chlamydia trachomatis* variant escaping detection by a widely used assay in Finland that was found in other European countries thereafter [15-17].

An activity close to our heart is the *Eurosurveillance* lunchtime seminar that is organised every year on the margins of the European Scientific Conference on Applied Infectious Disease Epidemiology (ESCAIDE). The 2019 seminar about Point-of-care testing (POCT) and its impact on surveillance of communicable diseases and public health was well received and we look forward to publishing a respective special issue sometime in early spring. The call for the next special issue on *Food as a vehicle for AMR* will be launched in the second half of 2020. Other 2019 activities by the *Eurosurveillance* editors comprised several workshops on publication ethics, tools to increase transparency in scholarly communications, and on how authors can improve their chances of getting published. Last but not least, we started to post educational article-related quizzes on Twitter.

All this could not have been achieved without the involvement of so many amazing supporters and constructive critics: our editorial board members, colleagues at the European Centre for Disease Prevention and Control (ECDC), public health experts and scientists in the field of infectious diseases and other disciplines in Europe and elsewhere who respond when we ask for assistance and who remain unnamed here. We owe you a heartfelt thank you! We also thank our publisher, the ECDC, and its Director who provide us with continued funding and editorial independence. We also value the contributions of our reviewers who go the extra mile, often on short notice and in their spare time, to help us select the right articles and guide authors in how they can improve their manuscripts. We are indebted to each of them. To acknowledge the ca 550 experts who reviewed for us in 2019, we are publishing a list with their names in this issue [18] and sent certificates to all those who supported us with more than one review.

In his 2020 New Year Message, United Nations (UN) Secretary-General António Guterres pointed out that we enter the new decade “*with uncertainty and insecurity”*, but he also stated that there is hope, addressing young people as “*the greatest source of that hope”* as they demand a role in shaping the future and encouraging them to challenge those in charge by “*speaking out”*, “*thinking big”*, “*pushing boundaries”* and “*keep[ing] up the pressure”*. He also noted that the UN will be launching a blueprint for further action for the sustainable development goals [19]. Also, the President of the European Commission Ursula von der Leyen’s agenda for Europe contains an “*aspiration* *of living in a natural and healthy continent”* and *a “world full of new technologies and age-old values”* [20]. Sustainability and evolution within scholarly communication are important to us editors. Together with our contributors and supporters, we are constantly working to maintain *Eurosurveillance* as a useful means to provide sound and trustworthy evidence for communicable disease prevention and control also in the future. In 2020, we will discuss with our editorial board our strategy and goals for the years 2021 to 2027; we envisage to further advance to follow new developments and meet the demands of our diverse audience to the best of our abilities also in the time to come. To get a better idea of the various needs and interests, we launch a satisfaction survey today. The results should inform our strategy by identifying areas of particular interest to our audience. We hope that many of you will respond and help us shape the future of the journal, so that *Eurosurveillance* continues to be interesting for readers, attractive for authors and useful for strategic and day-to-day public health decision-making.
